# Analysis of the dark proteome of Chandipura virus reveals maximum propensity for intrinsic disorder in phosphoprotein

**DOI:** 10.1038/s41598-021-92581-6

**Published:** 2021-06-24

**Authors:** Nishi R. Sharma, Kundlik Gadhave, Prateek Kumar, Mohammad Saif, Md. M. Khan, Debi P. Sarkar, Vladimir N. Uversky, Rajanish Giri

**Affiliations:** 1 School of Interdisciplinary Studies, Jamia Hamdard-Institute of Molecular Medicine (JH-IMM), Jamia Hamdard, Hamdard Nagar, New Delhi, 110062 India; 2 School of Basic Sciences, Indian Institute of Technology Mandi, VPO Kamand, Kamand, Himachal Pradesh 175005 India; 3 Department of Biochemistry, University of Delhi South Campus, New Delhi, 110021 India; 4 Department of Molecular Medicine and Byrd Alzheimer’s Research Institute, Morsani College of Medicine, University of South Florida, Tampa, FL 33620 USA; 5 Institute for Biological Instrumentation of the Russian Academy of Sciences, Federal Research Center “Pushchino Scientific Center for Biological Research of the Russian Academy of Sciences”, Pushchino, 142290 Moscow Russia

**Keywords:** Protein analysis, Protein sequence analyses, Biochemistry, Biophysics, Computational biology and bioinformatics, Molecular biology, Structural biology, Diseases, Molecular medicine

## Abstract

Chandipura virus (CHPV, a member of the *Rhabdoviridae* family) is an emerging pathogen that causes rapidly progressing influenza-like illness and acute encephalitis often leading to coma and death of the human host. Given several CHPV outbreaks in Indian sub-continent, recurring sporadic cases, neurological manifestation, and high mortality rate of this infection, CHPV is gaining global attention. The ‘dark proteome’ includes the whole proteome with special emphasis on intrinsically disordered proteins (IDP) and IDP regions (IDPR), which are proteins or protein regions that lack unique (or ordered) three-dimensional structures within the cellular milieu. These proteins/regions, however, play a number of vital roles in various biological processes, such as cell cycle regulation, control of signaling pathways, etc. and, therefore, are implicated in many human diseases. IDPs and IPPRs are also abundantly found in many viral proteins enabling their multifunctional roles in the viral life cycles and their capability to highjack various host systems. The unknown abundance of IDP and IDPR in CHPV, therefore, prompted us to analyze the dark proteome of this virus. Our analysis revealed a varying degree of disorder in all five CHPV proteins, with the maximum level of intrinsic disorder propensity being found in Phosphoprotein (P). We have also shown the flexibility of P protein using extensive molecular dynamics simulations up to 500 ns (ns). Furthermore, our analysis also showed the abundant presence of the disorder-based binding regions (also known as molecular recognition features, MoRFs) in CHPV proteins. The identification of IDPs/IDPRs in CHPV proteins suggests that their disordered regions may function as potential interacting domains and may also serve as novel targets for disorder-based drug designs.

## Introduction

Chandipura virus (CHPV) was first isolated in 1965 in the Indian state of Maharashtra, from a patient suffering from the febrile illness, with the ability to produce cytopathic effect on cell culture^1^. CHPV is a member of the Genus V*esiculovirus* in the family *Rhabdoviridae*. Later it was also isolated from the encephalopathy patients in 1980^2^. However, the first evidence for the CHPV association with human epidemics was obtained in 2003, when this virus was identified in patient samples during an outbreak in India as a determinant of the acute encephalitis with a high fatality rate claiming 183 lives, mostly children below the age of 12^3^. The medical examination of patients recorded high-grade fever, occasional vomiting, rigours, sensorium, drowsiness leading to coma and death within 48 h. Subsequently, another outbreak of CHPV infection with more than 75% fatality rate was reported in the eastern region of Gujarat, India, in 2004^4^. These recurrent occurrences indicated possible emergence of CHPV as a deadly human pathogen in the Indian subcontinent causing acute encephalitis syndrome and involving severe human pathology, which progresses rapidly from an influenza-like illness to coma and death^3^. The female sandflies (*Phlebotomine sandfly*)^5^, ticks^6^, and mosquitoes^7^ are proposed to be the CHPV carriers, wherein this arthropod virus resides in the salivary gland of these insects and is transmitted to the mammalian host through bites. While the route of CHPV migration to CNS remains unclear, its neurotropic ability was established in suckling Balb/c mouse pups injected with CHPV through their footpads and in adult mice infected through intracerebral route, upon which progressive viral replication in spinal cord and brain of sucking mice and in brain of adult mice was observed^8^.

While prevalent in tropical and subtropical regions, CHPV poses a serious threat to public health in the entire Indian subcontinent. Notably, with an increase in travel and globalization, viruses are no longer restricted to national boundaries. Furthermore, CHPV detection in sandflies of African continent^9^ forecast the high risk of its spread causing an epidemic in more parts of the globe. Given these forewarnings, it is of paramount importance to comprehensively understand CHPV biology and make efforts in the direction of developing antiviral measures. The lack of any vaccine or effective treatment against this virus stresses the immediate and urgent need for finding and developing corresponding antiviral therapeutics.

**Figure 1:**
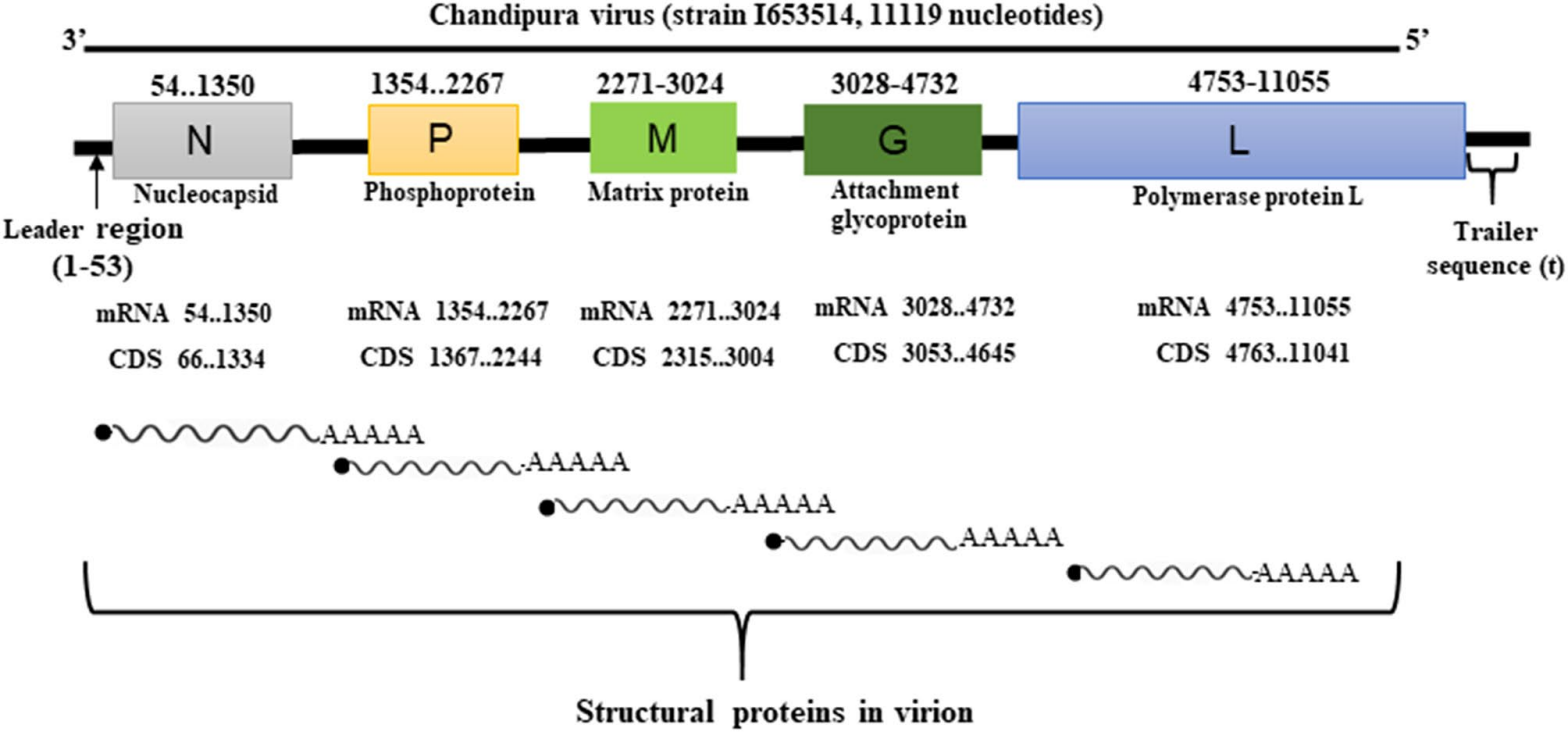
Genome architecture of CHPV. It contains genes for Nucleoprotein (N), Phosphoprotein (P), Matrix protein (M), Glycoprotein (G), and RNA-directed RNA polymerase L (L).

The ~ 11.119 kb CHPV genomic RNA (11,119 nucleotides, nts) contains a 49 nt leader gene (l), five transcriptional units coding for viral polypeptides arranged in the order 3′ l-N-P-M-G-L-t 5′ separated by spacer regions and followed by a short non-transcribed 46 nt trailer sequence (t) (Fig. 1). Following partial sequencing of its (+) leader RNA^10^, N and P gene^11^, full-length genome was obtained recently^12^. Comparative sequence analysis projected CHPV to be evolutionary central from the New World vesiculoviruses VSV or vesicular stomatitis virus Indiana and VSV New Jersey (VSVnj) and rather closely related to the Asian vesiculovirus Isfahan^12^.

The shape of CHPV resembles a typical bullet, which is 150–165 nm long and 50–65 nm wide, as determined by transmission electron microscopy^6^. It is an enveloped virus with a helical ribonucleoparticle (RNP) surrounded by an outer bilayer lipid membrane. Glycoprotein G protrudes externally from the outer membrane, while Matrix protein M lies inward of the inner leaflet of the outer membrane. The RNP containing its genomic RNA is enwrapped by the virally encoded N (nucleocapsid) protein^13^. Besides N, the other two viral proteins, L and P, are also packaged within the mature virion, being associated with core nucleocapsid particles, and serve as two components of viral RNA dependent RNA polymerase.

Intrinsically disordered proteins (IDPs) exhibit specific functions without being folded into a unique 3D structure under physiological conditions^14–17^. In most systems, IDPs representing the dark proteome, range from fully unstructured proteins to a hybrid containing IDP regions (IDPRs) as well as structured regions. Intrinsic disorder (ID) phenomenon is highly heterogeneous and includes random coils, molten globules, and disordered/flexible regions (e.g., flexible linkers connecting domains in large multi-domain proteins)^18^. IDPs/IDPRs were shown to have a wide range of implications in human diseases including cancer and neurodegenerative disorders^19, 20^. More than 30% of the human proteome is believed to be comprised of IDPs/IDRs which remain hidden during structural characterization that utilizes the traditional structural biology techniques, such as X-ray crystallography^21^. The unique characteristics of IDP sequences have led to the development of various algorithms for rather accurate disorder prediction. Utilization these predictors suggested high abundance of ID in nature, with many proteins being disordered along their entire length. Besides charge and amino acid polarity, the hydrophobic interactions play a major role in energetically favoring the protein folding^22^. The analysis of disordered or unstructured regions showed compositional bias in their amino acid sequences, with a significantly larger proportion of small, charge, and polar amino acids and proline residues and noticeably depleted content of hydrophobic residues than those found in structured regions^23^. IDPs can feasibly adopt a fixed three-dimensional structure upon binding to other macromolecules (at least in parts engaged in direct interaction), thereby showing the capability to undergo binding-induced disorder to order transitions. Interestingly, many IDPs/IDPRs, for example, transactivation domain of c-Myb, show disorder to order transition by attaining an α-helical conformation after binding to its partner KIX^24^. Furthermore, reports also suggested that a single mutation in IDPRs may change their structural propensity^25^. Notably, many viral proteins possess molecular recognition feature (MoRF) regions, which are short regions in IDPs that undergo a disorder-to-order transition upon binding to their interacting partners. Structural and non-structural proteins of Zika virus have MoRF regions that regulate the functionality of this virus^26^. It is now acknowledged that IDPs/IDPRs not only play a vital role in the formation of several macromolecular complexes^27^ but also participate in the assembly of RNA and proteins to form RNA granules^28^. Furthermore, it is recognized now that disordered regions represent new and attractive targets for drug designs^29–32^.

Intrinsic disorder in proteins facilitates their interaction with many biological partners and thus constitutes an important prerequisite for proteins to serve as hubs in protein–protein interaction networks regulating multiple cellular pathways^33–35^. Bioinformatics analysis has shown prevalence of the intrinsic disorder in various viral proteins^36–40^. The large IDPRs in viral proteins can be indispensable for the various functioning of these proteins, for example for adaptation, accommodation of the virus in hostile habitats, helping the virus in the proper management of its genetic material and also in the invasion of the host cell pathways^41, 42^. In this study, we have employed a set of bioinformatics tools to analyze the propensity of the proteins of CHPV for intrinsic disorder, thereby categorizing ‘dark proteome’ of this virus. We also evaluated disordered regions in viral proteins in terms of their functional significance.

## Results and discussion

### Intrinsic disorder in CHPV proteome

**Figure 2:**
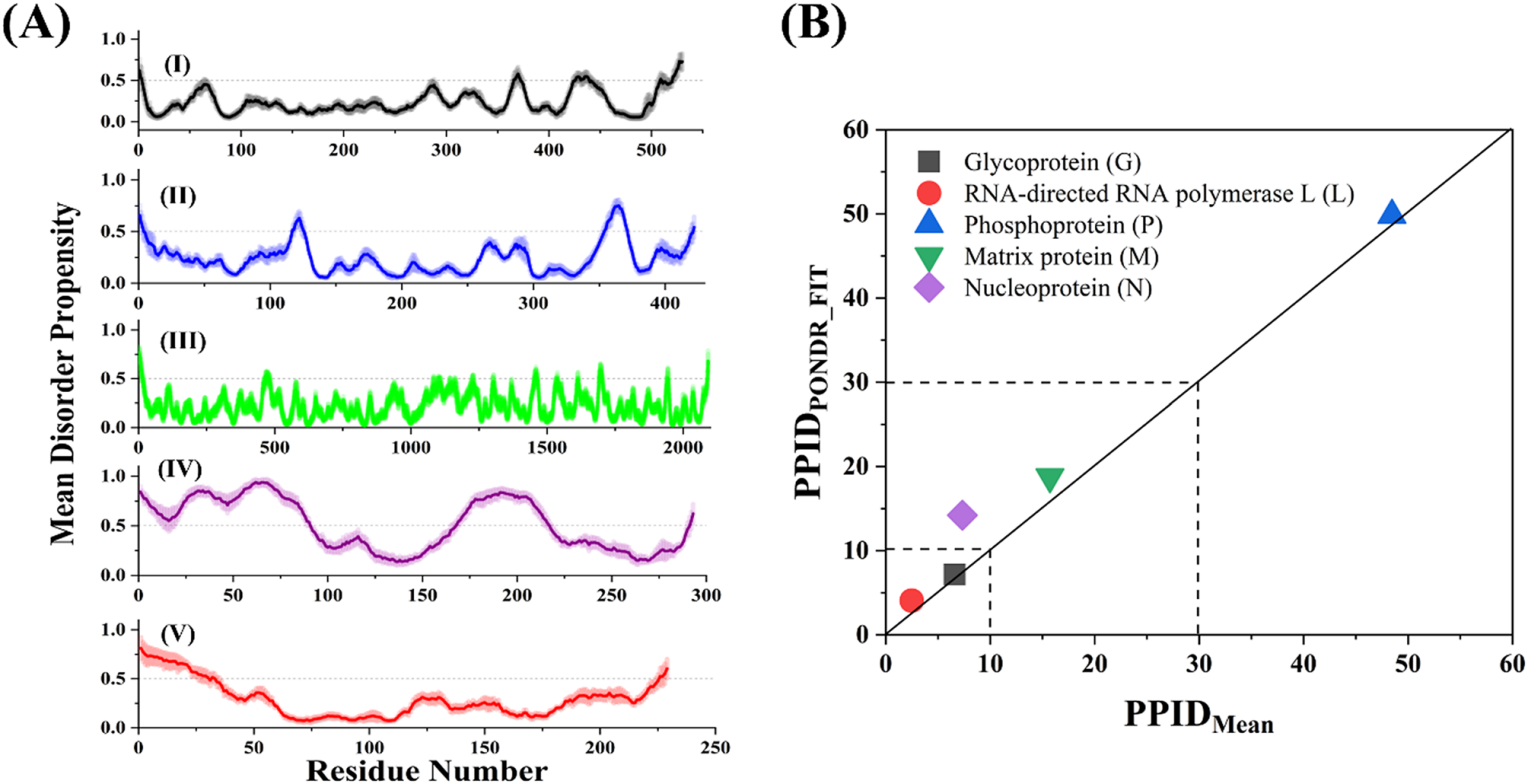
Evaluation of overall disorder status of five proteins of CHPV. (**A**) Mean disorder tendency with standard error (highlighted on the plot) for all five proteins of CHPV, (I) G, (II) N, (III) L, (IV) P, and (V) M. (**B**) 2D disorder plot represents the PPID_PONDR-FIT_ vs. PPID_mean_ dependence.

We performed intrinsic disorder predisposition analysis of proteins from CHPV proteome (Table 1). The genome of CHPV codes for five polypeptides, namely, Nucleocapsid protein N (422 residues), Phosphoprotein P (293 residues), Matrix protein M (229 residues), Glycoprotein G (530 residues), and Large protein L (2092 residues) in five monocistronic mRNAs (Fig. 1). Figure 2A through E represent the disorder profiles for each of the CHPV protein calculated as mean from all seven disorder predictors utilized in this study. Further, to get a global overview of the disorder status in these proteins, we looked at the PPIDs (predicted percent of intrinsic disorder) in these proteins evaluated by PONDR FIT (PPID_PONDR-FIT_) and mean PPIDs (PPID_mean_) of these proteins. Results of this analysis are shown in Fig. 2B that represents 2D-disorder plot; i.e., the PPID_PONDR-FIT_ *vs*. PPID_mean_ plot. According to the overall levels of intrinsic disorder, the proteins differentiates as highly ordered (PPID score between 0 and 10%), moderately disordered (PPID score between 10 and 30%), and highly disordered (PPID score more than 30%)^43^. The results clearly show that phosphoprotein is highly disordered; matrix protein and nucleoprotein are moderately disordered; and Glycoprotein G and Large protein L are highly ordered proteins. Although nucleoprotein PPID from seven different predictors are showing it ordered protein (Table 1), 2D-disorder plot places it to the group of moderately disordered proteins.

**Table 1:**
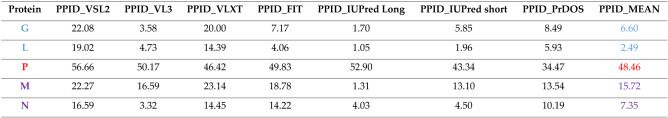
Intrinsic disorder in structural proteins of CHPV. Protein names and their mean PPIDs are colored (highly disordered- red, moderately disordered- purple, and ordered- light blue) to show their disorder status.

Among the five proteins expressed during CHPV infection, the crystal structure of only the ectodomain of CHPV-G protein is reported in PDB (PDB IDs: 4D6W and 5MDM). While the 3D structures of other CHPV proteins relying on the analysis of the scattering patterns of X-rays (X-ray crystallography), which reads electron density maps to understand protein 3D structure^44^, remains awaited, the use of computational analysis to observe disordered regions of the query protein may offer great advantages^45^. In addition, we have also analysed the sequence of Chandipura virus with its closely related family member Vesicular Stomatitis Indiana Virus (VSIV) using multiple sequence alignment (MSA).

IDPs/IDPRs are highly flexible and therefore can serve as a major reason for the inability of a protein to be crystallized or a reason for the lack of specific electron densities in X-ray structures. Our analysis using a set of specialized but commonly used predictor tools of the IUPred2^46^ and PONDR family shows that all CHPV proteins contain IDPRs. This disorder tendency varies among the proteins (Fig. 2, Table 1), with the highest mean PPID being obtained for P (48.46%) and M (15.72%) proteins, as compared to other proteins encoded by CHPV (Table 1).

In addition to intrinsic disorder, we have also computationally estimated the presence of disorder-based binding sites, MoRFs, in each of the five CHPV proteins. The MoRFs for individual proteins, identified by using four different computational tools (MoRFCHiBi_Web (MCW), MoRFpred, DISOPRED3, and ANCHOR), are listed in Table 2. All the proteins contain several MoRFs, representing their high binding promiscuity and profound predisposition for protein–protein interactions. The MCW is a meta-predictor and its predictions are fast and highly accurate for MoRFs predictions^47^. Hence, we have shown the MoRFs regions identified by MCW server 3D structures of G, P, and M proteins (see figures of individual proteins). MCW server does not recognize any MoRF in L and N proteins (Table 2).

**Table 2:** Identified MoRF regions in CHPV proteins.

Protein	MoRFCHiBi_Web (cutoff = ≥ 0.725)	MoRFpred (cutoff = ≥ 0.5)	DISOPRED3 (cutoff = ≥ 0.5)	ANCHOR (cutoff = ≥ 0.5)
G	_516_FEMRIFKPNNMRAR_529_	_382_WTQWFK_387_, _522_KPNNMRARV_530_	_1_MTSSVTISVVLLISFITPLY_20_, _485_VLIYGVLRCFPVLCTTCR_512_, _516_FEMRI_520_, _524_NNMRARV_530_	–
L	–	_93_AEWML_97_,_493_ATNWLEF_499_, _2084_FIESEHW_2090_	_1_MDLNPVDDAAELSEEN_16_, _2081_KTEFIES_2087_	–
P	_1_MEDSQLYQALKNYPKLQDTLDSIENLE_27_, 41TERGIPSYYLAEELD55, 278IYNRIRIR285	_10_LKNYPKL_16_,_43_RGIPSYYLAEEL_54_	–	_1_MEDSQLYQLKNYPKLQDLDSIENLEDDTKSEPSE_36_, _38_GSPTERGIPSYYLAEELDECEEEDSEEDDDNLPTEIPDPPTVDMLEAIMEDEIDDTAYQVHFEAKQT_104_, _214_APANLI_219_
M	_1_MQRLKKFIAKREKGDKGKMKWNSSMDYD_28_, _42_PTAPLF_47_	_2_QRLKKFI_8_	_1_MQRLKKFIAKREKGDKGKMKWNSSM_25_	–
N	–	_137_TLIFG_141_, _376_VVVWLAWWED_385_, _414_AEYARK_419_	_366_NDTTP_370_	–

### Intrinsic disorder in Glycoprotein (G)

Glycoprotein, G (UniProtKB ID P13180: GLYCO_CHAV, 530-amino-acid-long protein with the molecular mass of 59.185 kDa) is CHPV’s single spike protein that protrudes out from the viral lipid bilayer membrane and plays an essential role in virus attachment to the cellular receptor, assembly and budding of virion particles. While cellular receptor (s) for entry is yet to be known for CHPV, the single-pass transmembrane G protein is believed to mediate receptor binding and catalyse membrane fusion in order to gain entry to the host cell. The CHPV G protein consists of an N-terminal signal peptide (residues 1–21) followed by three domains, such as an ectodomain (residues 22–473), transmembrane region (residues 474–494), and a cytosolic domain, (residues 495–530). A mature G protein acts as a major antigenic determinant and thus can induce the production of neutralizing antibodies^48^. Expression of the *G* gene in COS cells resulted in the production of a glycosylated protein of molecular weight 71,000 daltons, which was recognized by anti-Chandipura antibodies^49^. The comparison and sequence alignment with other rhabdoviruses proposed two putative sites (184 and 344; as per Uniprot database) for glycosylation in the G protein of all CHPV isolates^50^. Sequence analysis among the CHPV isolates showed that G gene is less conserved (with 7–11 amino acid changes) compared to genes encoding N or P proteins showing more than 95–97% homology, respectively^51^. The GFPP motif of the CHPV G protein is involved in viral fusion with host cell membrane^52^, and a comparative analysis of the whole genomes of CHPV isolates with other rhabdoviruses showed that this motif is conserved at position 129 in all CHPV isolates as in other vesiculoviruses^50^. Interestingly, all amino acid substitutions in G protein sequence were found in the ectodomain^51^. Based on sequence alignment of CHPV G protein with its closely related VSIV G protein, nearly 40% of sequence similarity exists within these two sequences (Supplementary Figure S1).

**Figure 3:**
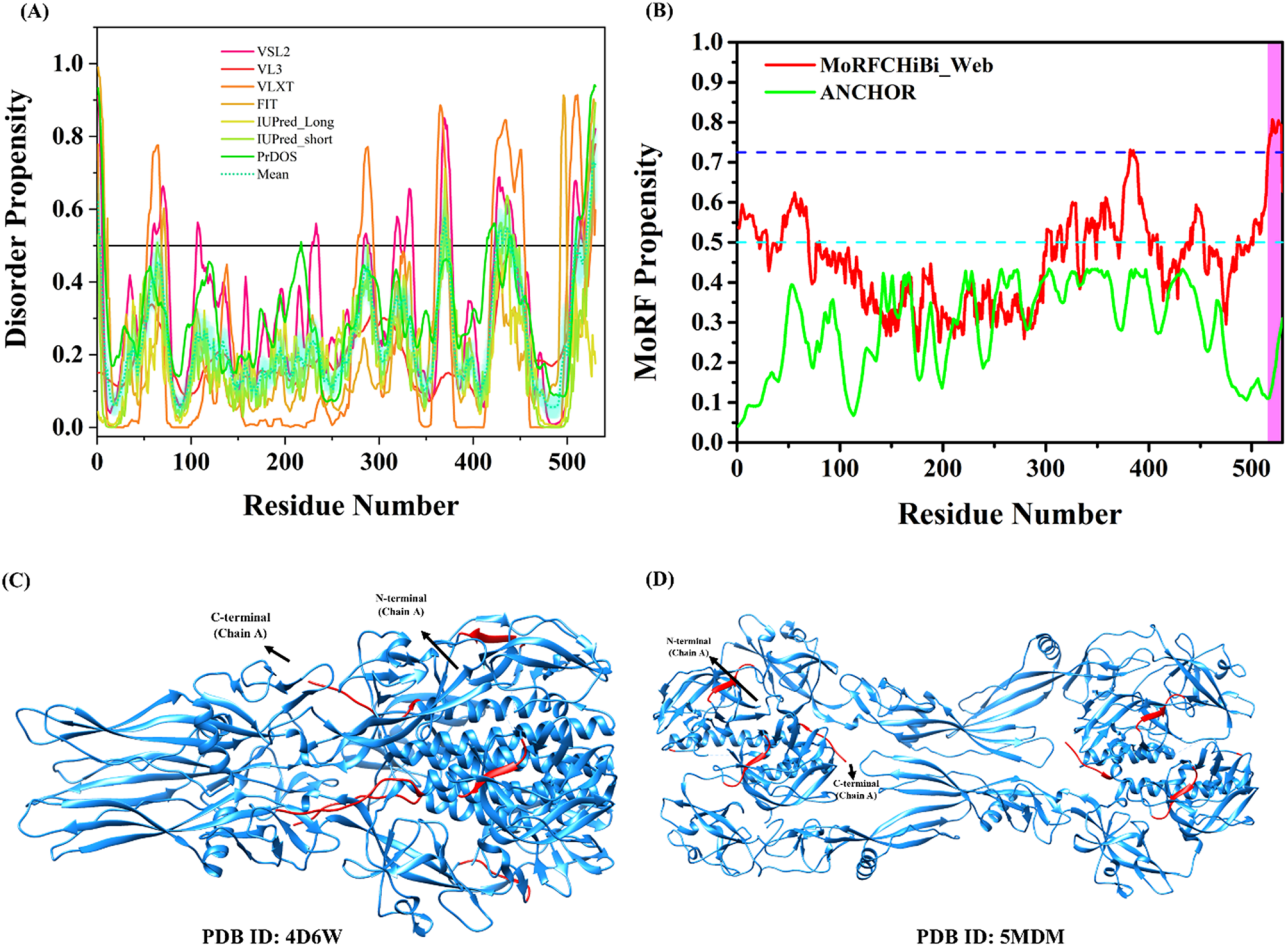
Intrinsic disorder predisposition of Glycoprotein (G). (**A**) The intrinsic disorder profile generated for Glycoprotein by a set of disorder predictors; PONDR VSL2, PONDR VL3, PONDR VLXT, PONDR FIT, IUPred2_long, and IUPred2_short are represented by black, red, blue, magenta, dark yellow-, and navy-colored straight lines respectively. A mean disorder profile calculated by averaging the outputs of seven predictors is represented by the green-colored short-dash line. Light green region around mean curve represents the error distribution for the mean. (**B**) Plot of MoRFs prediction by MCW and ANCHOR. Dashed cyan line (0.5) represents cut-off for ANCHOR and dashed blue line (0.725) represents cut-off for MCW server. The area with light magenta color represents MoRF region predicted by MCW server. (**C**) PDB ID: 4D6W. (**D**) PDB ID: 5MDM. Red colored regions are predicted to be disordered based on the calculated mean. The N- and C-terminals are shown with arrows for chain A in both structures.

In our disorder prediction-based analyses, the protein is characterized by a mean PPID of 6.6%, as calculated based on the outputs of seven intrinsic disorder predictors used in our study (Fig. 3A). Moreover, the MobiDB has predicted the Glycoprotein to be fully ordered based on the consensus of different predictors. It is possible that the IDPRs identified in our study provide the flexibility to G protein required in the fusion process. Furthermore, we looked for the presence of disorder-based interaction sites in CHPV G protein using four specialized predictors and found several unique overlapping MoRFs regions (residues 1–20, 382–387, 485–512, 516–520, 516–529, 522–530, and 524–530). MCW server predicted one MoRF region (residues 516–529) at the C-terminal end of the G protein (Table 2, Fig. 3B), DISOPRED3 predicted multiple MoRFs regions (residues 1–20, 485–512, 516–520, and 524–530), whereas MoRFpred predicted two MoRFs regions (residues 382–387 and 522–530) (Table 2).

Vesiculoviruses entry to the host cells occurs through membrane fusion, induced by a conformational change in the fusion glycoprotein G provoked by low pH environment. This conversion involves transition from a trimeric pre‐fusion toward a trimeric post‐fusion state via monomeric intermediates. The crystal structure of the CHPV glycoprotein G soluble fragment (1–419) obtained after proteolysis with thermolysin, in the low pH-induced post-fusion conformation was determined with a resolution of 3.6 Å (Fig. 3C)^53, 54^. Another crystal structure of the CHPV G protein at intermediate pH with a resolution of 2.998 Å showed two intermediate conformations forming a flat dimer of heterodimers (Fig. 3D)^55^. These studies revealed the range of G structural changes and suggested that G monomers can re‐associate, through antiparallel interactions between fusion domains, into dimers that play a role at an early stage of viral-host cell fusion process.

Our analysis revealed that in the G protein, several ID regions exist all along the protein. The N-terminal region of G protein which contain fusion peptide (116–137aa) with a GFPP motif in VSV, is mainly ordered. The membrane-proximal C-terminal region of the ectodomain has most of its intrinsic disorder with residues 366–372, 426–439, and 521–530 forming IDPRs. This membrane-proximal region was demonstrated to be critical for the viral fusion and virus infectivity in several viruses, including VSV Glycoprotein G ectodomain^56^. Experimentally determined crystal structures have total residue length of 1–419 residues. Hence, predicted IDPRs (residues 426–439 and 521–530) could not be mapped in the structure (Fig. 3C,D). A short stretch of predicted disordered residues (amino acids 366–372) fall in the β-structured region, forming a loop. However, these regions might possibly be flexible in nature.

### Intrinsic disorder in Nucleoprotein (N)

The Nucleocapsid protein (N) (UniProtKB ID: P11211; NCAP_CHAV, a 422-amino-acid-long polypeptide with a molecular mass of 47.9 kDa) of CHPV is the most abundantly expressed viral protein in the infected cells^57^. It plays several crucial roles in the viral life cycle, besides being a vital structural component of the virion by proper organization of its interactions with other viral components^57^. However, the major function of CHPV N protein is to enwrap the viral RNA and protect it from degradation by cellular RNases. CHPV N gene shares nearly 50.6% identity with the N protein of VSV, its closest neighbour (Supplementary Figure S2).

**Figure 4:**
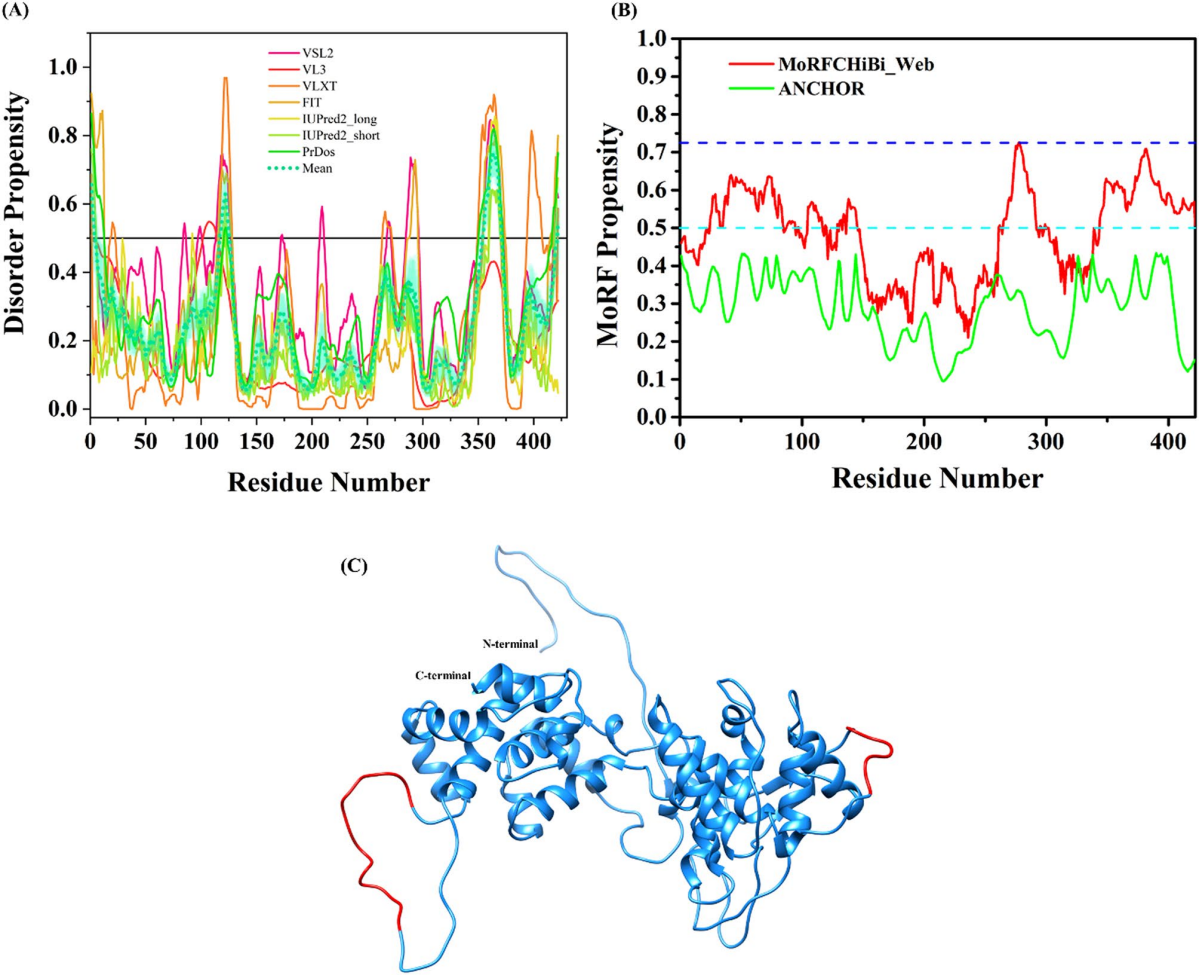
Intrinsic disorder predisposition of Nucleoprotein (N). (**A**) The intrinsic disorder profile generated for Nucleoprotein by a set of disorder predictors; PONDR VSL2, PONDR VL3, PONDR VLXT, PONDR FIT, IUPred2_long, and IUPred2_short are represented by black, red, blue, magenta, dark yellow-, and navy-colored straight lines respectively. A mean disorder profile calculated by averaging the outputs of seven predictors is represented by the green-colored short-dash line. Light green region around mean curve represents the error distribution for the mean. (**B**) MoRFs prediction through MoRFCHiBi_Web and ANCHOR. Dashed cyan line (0.5) represents cut-off for ANCHOR and dashed blue line (0.725) represents cut-off for MCW server. The area with light magenta color represents MoRF region predicted by MCW server. (**C**) Full-length modelled structure for N protein using I-Tasser web server. Red colored regions are predicted to be disordered based on the calculated mean. The N- and C-terminals are shown with arrows in the structure.

Our disorder analysis revealed that in the N protein, two regions (residues 117–125 and 355–371) of intrinsic disorder are present. The protein is not disordered as predicted by MobiDB as well as it has given an overall mean PPID of 7.35% calculated from the output of seven predictors used in this study (Fig. 4A, Table 1). It appears from these data that N protein is ordered up to a great extent however the C-terminal lobe has few regions with intrinsic disorder property. The extended loop (residues 340–375) is found to be intrinsically disordered (residues 355–371) and seems to have implications in the RNA binding ability of this protein. The N-lobe (residues 110–130) is also found to be disordered (residues 117–125) that may be important for ability of this region to bind P protein. Furthermore, the MoRFs analysis using four different predictors located several MoRFs within the N protein (residues 137–141, 366–370, 376–385, 414–419) (Fig. 4B, Table 2). Of these, DISOPRED3 predicted one MoRF region (residues 366–370), while MoRFpred predicted three MoRFs regions (residues 137–141, 376–385, 414–419). These data suggested the presence of disorder-based protein binding regions at the C-terminal lob of the N protein.

The crystal structure (2.9 Å) of VSV-N was obtained^58^ in a complex containing 10 molecules of the N protein and 90 bases of RNA tightly sequestered in a cavity at the interface of two lobes of the N protein. These two lobes found in the crystal structure of VSV-N contain mainly α helices, which come together to form a cavity that accommodates RNA. The N-terminal lobe contains seven α-helices along with four β-strands, while the C-terminal lobe beginning at residue Ser220 contains eight α-helices. Besides these lobes, an N-terminal arm (residues 1–22) containing two anti-parallel β-strands and a C-terminal extended loop (residues 340–375) was also shown to be important for the N oligomerization and RNA binding^58^. The encapsidation of replication products by VSV-N protein is concurrent with genomic RNA synthesis forming a precise structure^10, 59–61^. This encapsidation is proposed to protect the RNA from degradation in the absence of polynucleotide synthesis. Based on these crystallized structures of VSV-N (PDB ID: 3HHZ, 2GIC, 3HHW), we built a 3D model of full-length CHPV-N protein using I-Tasser web server. The obtained model is shown in Fig. 4C, depicting N- and C-terminals and identified disordered residues with red color.

Although N protein displays broad RNA sequence specificity that is consistent with the observed mode of RNA binding in crystal structure, proper initiation of the encapsidation entails definite recognition of the sequence elements present at the genome termini^10, 60, 61^. The N protein plays a dual role by its ability to recognize specific sequence on nascent RNA, known as nucleation. In its monomeric state, N recognizes a specific sequence within the first 21 nucleotides of the leader RNA, which is not recognized by the oligomerized N protein. During the nucleation step, N monomer initiates nucleocapsid assembly on nascent viral leader RNA^62^. During elongation phase, the N–N association results in both inter- and intracellular conformational changes that enable the newly polymerized N protein to bind to the heterogeneous sequence on the RNA molecule, while the N–P complex provides continued N monomers.

While VSV-N prepared in the soluble form showed the tendency to aggregate and to assemble with leader RNA in a sequence-dependent manner^10^, its ectopic expression in the eukaryotic cells also showed cytosolic aggregates^63^. As demonstrated in CHPV, this tendency to self-associate is completely abrogated upon deletion in the N-terminal arm, whereas the C-terminal 102 residues are important for specific recognition of the viral leader RNA^57^. Using deletion mutants it was shown that the N-terminal 47 amino acids together with residues 180–264 are indispensable for the N protein oligomerization^57^. It is the interaction of monomeric N protein with phosphoprotein (P), which maintains N in the encapsidation competent soluble (active) form^64, 65^. Within the VSV infected cells, N–P complexes of varying molar ratios were observed^66, 67^.

Earlier performed CHPV analysis mapped interacting viral proteins, such as N–N and N–P, to the domain level^57, 68^. The N-terminal 180 residues and the C-terminal 102 residues of N protein are required for binding to P protein in its monomeric and RNA-encapsidated state, respectively^68^. A different study using yeast two-hybrid and ELISA revealed the unique binding site consist of residues 1–30 at the N terminus of the nucleocapsid protein (N1) involved in its interactions with N, P, M, and G proteins. It was also observed that N2 fragment (a 278-residue-long internal fragment overlapping with the 10 residues from N1 and 68 residues from C-terminal domains) associated with N and G proteins while C-terminal 193-residue-long N3 fragment interacts with N, P, and M proteins^69^.

### Intrinsic disorder in RNA-directed RNA polymerase L (L)

The L protein (UniProtKB ID: P13179;L_CHAV, a 2092-amino-acid-long polypeptide with a molecular mass of 238.5 kDa) and P protein together constitutes viral RNA-dependent RNA polymerase. In this complex, L protein retains the catalytic activity of RNA polymerization, as well as capping and polyadenylation functions, and P acts as a transcriptional activator. CHPV L protein exhibits a high degree of homology with its counterparts in other rhabdoviruses. The conserved residues in VSV are also present in CHPV-L protein^70^, with a central region^12^ being responsible for RNA polymerization. The overall similarity between both sequences of CHPV and VSV is 59% (Supplementary Figure S3).

It has been demonstrated that the L protein of CHPV exhibits a VSV-like RNA : GDP polyribonucleotidyltransferase (PRNTase) activity, which transfers the 5′-monophosphorylated (p-) viral mRNA start sequence to GDP to produce a capped RNA, and that the conserved (histidine-arginine) HR motif in the CHPV L protein is essential for the PRNTase activity. A universal use of the active-site HR motif by rhabdoviral L protein for the PRNTase reaction at the step of the enzyme–pRNA intermediate formation was suggested^71^. Capping reactions catalyzed by L protein in VSV has evolved independent of eukaryotes. The L protein of VSV incorporates the GDP moiety of GTP into the cap structure of mRNAs instead of GMP as in eukaryotes^72^. The 5′ end modification events were proposed to be successive to transcription initiation, whereas the nascent mRNA termini maintain contact with the transcribing polymerase until being modified^73^. The addition of poly(A) tail to the viral mRNA is also attributed to the L protein, where polymerase slippage during transcription termination at U7 tract is believed to add A residues at the 3′ end of mRNA^74^. VSV L protein is also shown to be associated with protein kinase activity, whether intrinsic or due to cellular kinase, L associated kinase (LAK)^59, 75^. The translation elongation factor, EF1 is also found to be associated with L protein. It was speculated that EF1 is important for L activity as an RNA polymerase^76^. Altogether, L protein along with P protein and some specific cellular components synthesize viral mRNA within infected cells.

**Figure 5:**
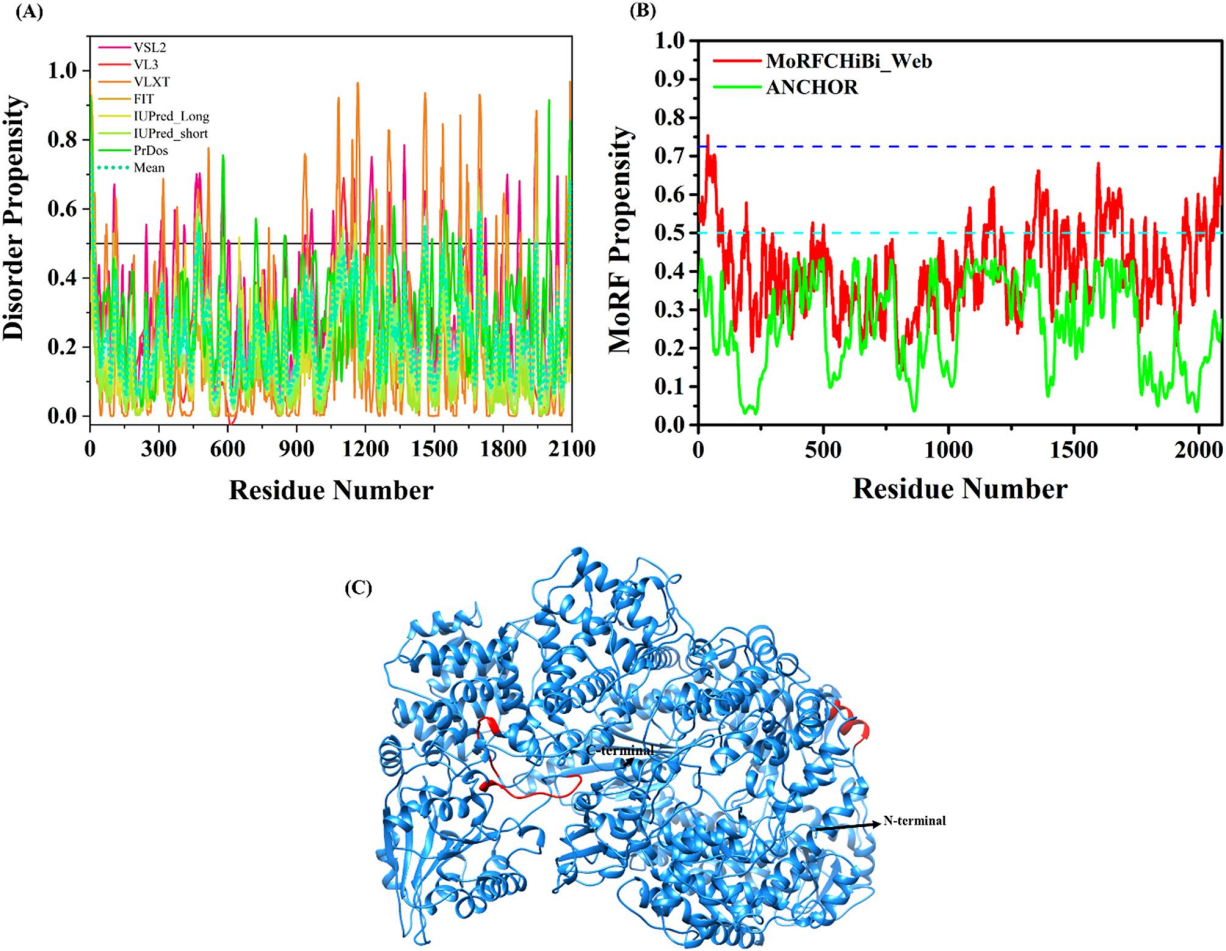
Intrinsic disorder predisposition of RNA-directed RNA polymerase L (L). (**A**) The intrinsic disorder profile generated for L protein by a set of disorder predictors; PONDR VSL2, PONDR VL3, PONDR VLXT, PONDR FIT, IUPred2_long, and IUPred2_short are represented by black, red, blue, magenta, dark yellow-, and navy-colored straight lines respectively. A mean disorder profile calculated by averaging the outputs of seven predictors is represented by the green-colored short-dash line. Light green region around mean curve represents the error distribution for the mean. (**B**) MoRFs prediction by MoRFCHiBi_Web and ANCHOR server. Dashed cyan line (0.5) represents cut-off for ANCHOR and dashed blue line (0.725) represents cut-off for MCW server. (**C**) Modelled structure for L protein (residues 32–2092) using Swiss Model. Red colored regions are predicted to be disordered based on the calculated mean. The N- and C-terminals are shown with arrows in the structure.

Our analysis showed that in the L protein, although being the largest proteins of CHPV, contains the lowest levels of intrinsic disorder compared to other CHPV proteins. The protein is characterized by lowest overall PPID of 2.49%, as calculated from the output of seven predictors of intrinsic disorder used in our study (Fig. 5A, Table 1), suggesting most of structure-functional relationship with respect to its functions. Four short disordered regions (residues 1–15, 466–474, 1454–1463, and 1691–1702) were identified in the L protein. However, the MobiDB consensus has not predicted to be disordered. The disorder-based binding regions or MoRFs analysis in CHPV L protein by a set of four specialized predictors collectively finds several short MoRFs at various regions (residues 1–16, 93–97, 493–499, 2084–2090, and 2081–2087) (Fig. 5B, Table 2). The MoRFpred server predicted three MoRFs regions (residues 93–97, 493–499, and 2084–2090) and DISOPRED3 predicted two MoRFs regions (residues 1–16 and 2081–2087). Further, to have the clearer picture of the order–disorder interplay in this protein, we constructed a homology model using L homologues from VSV in Swiss Model (Fig. 5C). Due to low homology within the N-terminal region, first 31 residues were not modeled. Out of four predicted disordered regions, three have been shown in the structure, while the N-terminal part is omitted.

### Intrinsic disorder in matrix protein (M)

The matrix protein M (UniProtKB ID: Q9WH76;MATRX_CHAV, a 229-residue long protein with the molecular mass of 26.6 kDa) is a multifunctional protein that is located in the inner surface of the virion to hold core nucleocapsid to the membrane and plays major role in virus assembly and budding, virus-induced inhibition of host gene expression, and cytopathic effects (including rounding of cells and apoptosis) observed in the infected cells. Like other CHVP proteins, most of the current understanding of how CHPV M protein functions are based on the earlier studies performed on the M protein from closely related VSV, which is also a vesiculovirus. For example, a motif PPPY in VSV was shown to be involved in the late stage of virus budding^77^. It was found that the N-terminus of M-protein of all the CHPV isolates contained this highly conserved PPSY (30–33) sequence also identified in other vesiculovirus, Isfahan virus^50^. While in VSV, eight lysine residues within the first 20 residues define a highly basic nature of the N-terminal domain and facilitate its membrane binding^78^, in CHPV, seven lysine residues in the N-terminal domain are present and can be proposed to mediate binding to membrane as well. However, in VSV, this domain separated from the rest of the polypeptide by a polyproline sequence (triplet)^79^, whereas CHPV does not seem to have this distinction. Also, the sequence similarity between M proteins of both viruses are quite less (29.3%) (Supplementary Figure S4).

A yeast two-hybrid system-based study identified ten host proteins interacting with CHPV M protein, three of which (CTD nuclear envelope phosphatase 1 (CTDNEP1), ATP-binding cassette sub-family E member 1 (ABCE1), and developmentally-regulated GTP-binding protein 1 (DRG1) were further validated by affinity pull-down and protein interaction ELISA^80^. The N-terminal 45 amino acids of CTDNEP1 behaves as a nuclear localization signal (NLS) and can target the bound protein to the nuclear membrane^81^. In the absence of any NLS in CHPV M protein, this interaction between the M protein and CTDNEP1 has been proposed to aid the viral protein to reach the nuclear membrane, where it is known to associate with the nuclear pore complex and subvert the nucleocytoplasmic transport of host mRNAs^80, 82^. This notion has been proven in several vesiculoviruses including CHPV that M protein inhibit nuclear transport of host mRNA and snRNA^83^ possibly by targeting nucleoporin Nup98 present on the nuclear rim, as shown in the case of VSV^82^. M protein regulated host gene expression inhibition is seen as an example of a viral mechanism to suppress cellular interferon response^84^. Since ABCE1 serves as the major source of energy during the assembly of viral capsids (e.g., HIV^85^, rabies virus^86^ and likely vesicular stomatitis virus^87^, interaction of this protein with CHPV M might provide support for the energy requirements needed for the formation of the characteristic bullet shaped virion of CHPV^80^.

**Figure 6:**
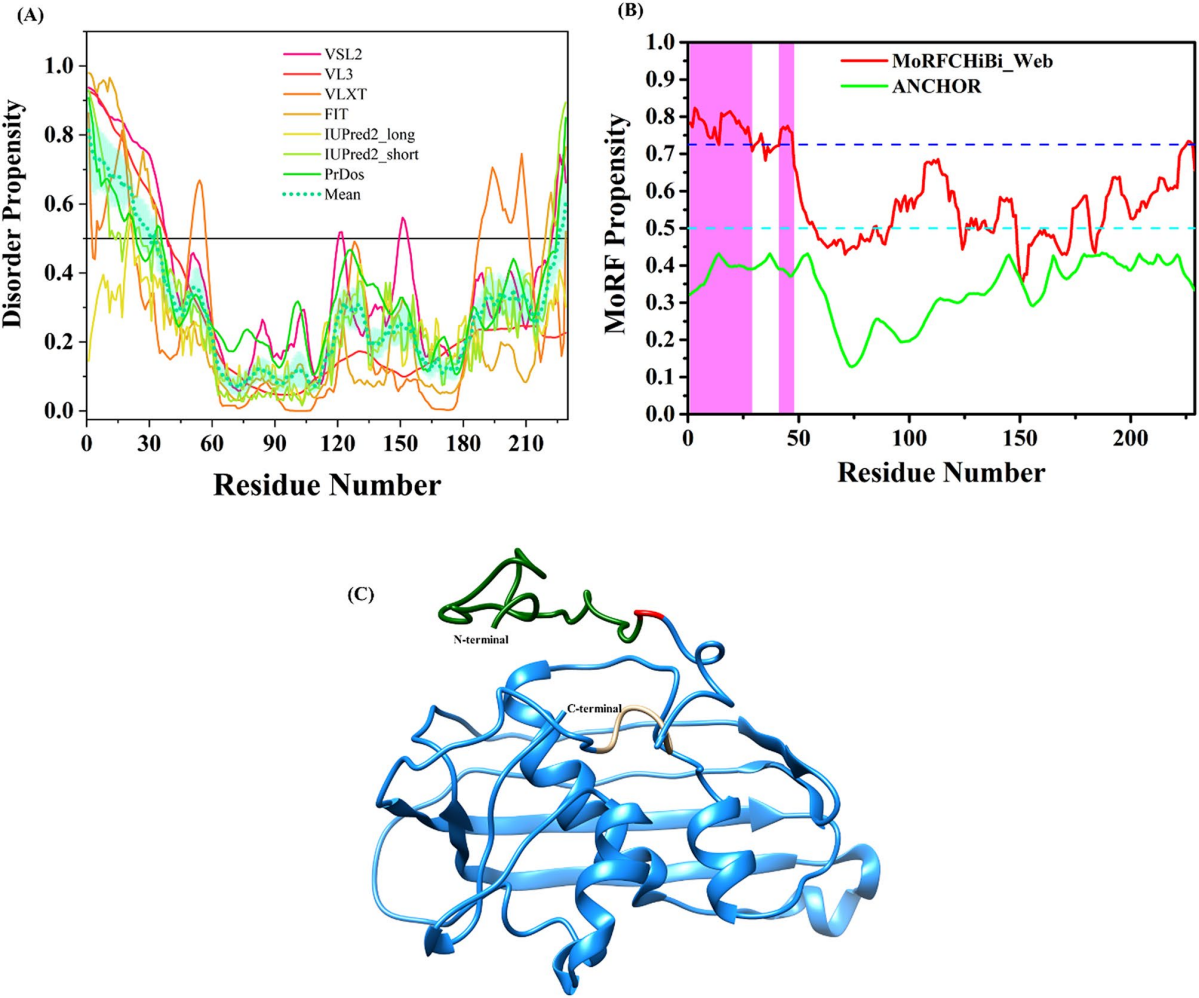
Intrinsic disorder predisposition of Matrix protein (M). (**A**) The intrinsic disorder profile for Matrix protein generated by a set of disorder predictors; PONDR VSL2, PONDR VL3, PONDR VLXT, PONDR FIT, IUPred2_long, and IUPred2_short are represented by black, red, blue, magenta, dark yellow-, and navy-colored straight lines respectively. A mean disorder profile calculated by averaging the outputs of seven predictors is represented by the green-colored short-dash line. Light green region around mean curve represents the error distribution for the mean. (**B**) MoRFs prediction by MoRFCHiBi_Web and ANCHOR server. The area with light magenta color signifies MoRFs region predicted by MCW server. Dashed cyan line (0.5) indicates cut-off for ANCHOR and dashed blue line (0.725) signifies cut-off for MoRFCHiBi_Web server. (**C**) Full-length modelled structure for M protein by I-TASSER web-server. The disordered (IDPRs), MoRFs residues and MoRFs in IDP predicted regions are shown in red, tan and green colors, respectively. The N- and C-terminals are shown in the structure.

Results of the intrinsic disorder predisposition analysis of the CHPV M protein are shown in Fig. 6. This analysis revealed that the N-terminal tail of the M protein is highly disordered (residues 1–30) and potentially serve as disorder-based protein binding region (Fig. 6A,B, Table 1). This indicates that intrinsic disorder and MoRFs have important role in functions of M protein and can be related to regulation of its nuclear localization via interaction with CTDNEP1.

While its X-ray crystal structure is awaited, our analysis revealed that the M protein is the second most disordered protein in CHPV proteome, with majority of its disorder being predicted within the N-terminal of the protein (residues 1–30). This region is located in the close proximity to the PPSY motif (residues 30–33) that plays a role in the virus assembly and budding during virus replication. The N-terminal IDPR might also be important for membrane binding properties of this protein, which were attributed earlier to eight lysine residues within the first 20 residues. While C-terminal of the protein is also predicted to have intrinsic disorder, the middle portion of the protein (residues 30–225) shows no disorder, suggesting the structure of this protein is dependent upon this region. The protein is characterized by an overall PPID of 15.72%, as calculated from the outputs of seven predictors of intrinsic disorder used in our study (Fig. 6A, Table 1). According to MobiDB lite, M protein does not contain any significant disorderedness and the consensus of other predictors has also predicted the same. Additionally, we checked for the presence of disorder-based binding regions in CHPV M protein, and four specialized predictors collectively found several MoRFs within the N-terminal region (residues 1–28, 42–47, 2–8, 1–25) of M protein (Fig. 6B, Table 2). The DISOPRED3 predicted one MoRFs (residues 1–25), while MCW identified two MoRFs regions (residues 1–28 and 42–47). MoRFpred predicted an overlapping region of seven amino acids (residues 2–8) of the two predictors (DISOPRED3 and MCW). These regions are shown in 3D model of the M protein structure built using I-TASSER (Fig. 6C). The server used two structures of matrix protein of VSV (PDB ID: 1LG7 and 2W2R) as templates to construct the model. As observed in the sequence-based disorder prediction, the N-terminal region is highly disordered and also contain MoRFs regions.

### Intrinsic disorder in phosphoprotein (P)

Phosphoprotein P (UniProtKB ID: P16380; PHOSP_CHAV) is a 293 amino acid protein with the molecular mass of 32.5 kDa. Together with CHPV L protein, P forms viral RNA-dependent RNA polymerase (RdRp), where it acts as a transcriptional activator. Although CHPV P protein show less than 20% similarity with P protein from other vesiculoviruses^11^, the reference for its phosphorylation can be obtained from studies on VSV, where cellular casein-kinase-II-induced phosphorylation state of P protein distinguishes the transcriptase and replicase action of RdRp^88, 89^. These studies demonstrated that VSV P protein functions as a transcription-replication switch, since the protein in its phosphorylated multimeric state (P1) forms a L-protein complex to construct functional transcriptase, while in its unphosphorylated state (P0), it interacts with L-protein to form replicase. However, the phosphoprotein has less similarity score (24.7%) among all other proteins with VSV proteins (Supplementary Figure S5).

The experimental evidence obtained for CHPV P corroborates with the phosphorylation-induced activity model of VSV. It has been shown that the unphosphorylated recombinant CHPV P protein expressed in *Escherichia coli* (BL21DE3) can be efficiently phosphorylated at Ser62 in vitro by casein kinase II (CKII), which induced dimerization and supported the transcription in vitro^90^. A mutant form of P protein with Ser62 replaced by alanine, being tested in vivo, could not trigger transcription and somewhat inhibited the viral mRNA synthesis trans-dominantly^91^. Therefore, the CKII-mediated phosphorylation seems to be essential for P protein to function as a transcription activator.

The N-terminal region of 46 amino acid was reported to be responsible for phosphorylation-mediated P-P homodimerization^92^. Here, the phosphorylation within the N-terminal region of the P protein was able to induce conformational changes in the protein leading to the transition from an ‘open’ to ‘closed’ structure. This phosphorylation-based structural alteration could change the accessible hydrophobic surface area of the protein and also the available digestion sites of different proteases. Biophysical experiments with the CHPV P protein showed that phosphorylation at Ser62 triggered a significant structural change in the N-terminal rgion of P protein, leading to exposure of the Cys57 residue to the protein surface^93^. Phosphorylation also resulted in the burying of tryptophan residues within the protein core while maintaining overall flexibility of N-terminal segment. Such conformational changes within the N-terminal domain of P were suggested to facilitate accurate polymerase contact with P1 to ensure optimal transcription^93^. Absence of such N-terminal phosphorylation in P can cause altered conformation and affect interaction with L-protein responsible for the formaton of a replicase complex^91, 94^.

The phosphorylation of P protein has also been shown to regulate its ability to bind to leader RNA, suggsting a possible role of this modification in genome transcription-replication switch^91^. Besides its role as a transcription-replication switch, the P protein also functions as chaperone in CHPV and plays a crucial role in the folding of nucleocapsid protein^90^. It binds via its C-terminus to N protein to maintains its soluble and active form that can encapsidate viral RNA. In VSV, the C-terminal domain of P protein was demonstrated to facilitate cooperative binding of multimeric phosphoprotein to polymerase (L) and template during transcription^95^.

**Figure 7:**
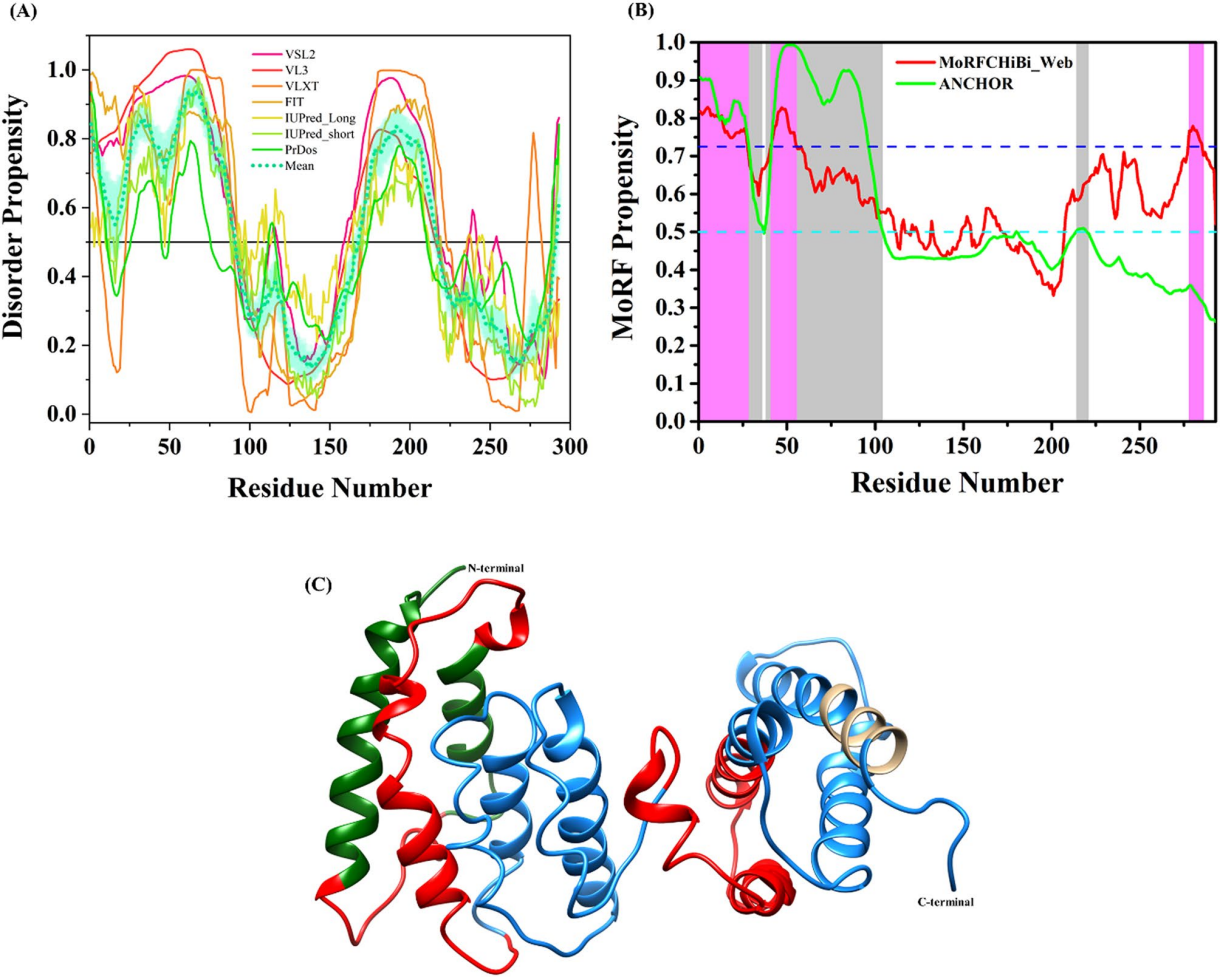
Intrinsic disorder predisposition of Phosphoprotein (P). (**A**) The intrinsic disorder profile generated for phosphoprotein by a set of disorder predictors; PONDR VSL2, PONDR VL3, PONDR VLXT, PONDR FIT, IUPred2_long, and IUPred2_short are represented by black, red, blue, magenta, dark yellow-, and navy-colored straight lines respectively. A mean disorder profile calculated by averaging the outputs of seven predictors is represented by the green-colored short-dash line. Light green region around mean curve represents the error distribution for the mean. (**B**) MoRFs prediction by MCW and ANCHOR server. The area with light magenta and light gray color signifies MoRFs region predicted by MCW and ANCHOR server, respectively. Dashed cyan line (0.5) represents cut-off for ANCHOR and dashed blue line (0.725) represents cut-off for MCW server. The area with light magenta color represents MoRF region predicted by MCW server. (**C**) Full-length modelled structure for P protein using I-TASSER web-server. The disordered (IDPRs), MoRFs residues and MoRFs in IDP predicted regions are shown in red, tan and green colors, respectively. The N- and C-terminals are shown with arrows in the structure.

Interestingly, computational analysis of phosphoprotein P revealed that this protein is the most disordered protein in the CHPV proteome. The protein is characterized by an overall PPID of 48.46%, which is calculated from the output of seven different predictors of intrinsic disorder (Fig. 7A, Table 1). Two continuous stretches of amino acids define two disordered domains (residues 1–90 and 168–217) of this protein. A stretch of 77 amino acids (residues 91–167) in between the two disordered domains and the C-terminal region, however, show potential presence of ordered domains in these regions of the protein. Also, MobiDB lite has also predicted residues 22–47, 55–74, and 171–211 of P protein to be disordered. It may be hypothesized that these predicted IDPRs have roles in the activity of phosphoprotein P as a transcription-replication switch. It might be interesting to investigate whether these disordered domains through phosphomodifictions act as regulators of P protein activity in the replication or transcription process. It may be possible that phosphorylation acts as a trigger for these disordered domains to convert into transactivation domains supporting their binding to their respective targets for its differential activity as a replication or transcription activator. Besides, our MoRF analysis in CHPV P protein identified numerous disorder-based protein binding regions within different parts of the protein (in fact, according to four computational tools used in our study, MoRFs can be found at residues 1–27, 10–16, 1–36, 38–104, 41–55, 43–54, 214–219, 278–285) (Fig. 7B, Table 2). MCW predicted three regions (residues 1–27, 41–55, and 278–285), MoRFpred predicted two regions (residues 10–16 and 43–54), and ANCHOR predicted three MoRFs regions (residues 1–36, 38–104, and 214–219). Of these, three predictor MCW (residues 1–27), MoRFpred (residues 10–16), and ANCHOR (residues 1–36) predicted a common/overlapping MoRFs region.

The longest stretch of 67 amino acids for disorder-based binding region (residues 38–104) was predicted by ANCHOR server. These results indicate that most of the disordered and disorder-based protein binding sites are located within the N-terminal half of the P protein that may have crucial role in phosphorylation-mediated P–P homodimerization. Due to less or no sequence similarity with structures in PDB, the threading approach of structure modelling based, I-Tasser web server used various structures to build the model (Fig. 7C). It also used a solution NMR structure of C-terminal region of VSV Phosphoprotein (PDB ID: 2K47). The sequence-based disorder analysis portrayed several residues to be disordered but the modeled structure has shown large ordered regions. However, these regions also constitute some short and distorted helical regions with less propensity which could lose their helical propensity. From the above analyses, it can also be interpreted that due to high disorderness, the structure could not be determined using experimental techniques. Hence, we have performed molecular dynamics (MD) simulation-based study on the modeled structure to determine its dynamics in real-time.

### Investigation on disorderness of phosphoprotein through MD simulations

**Figure 8:**
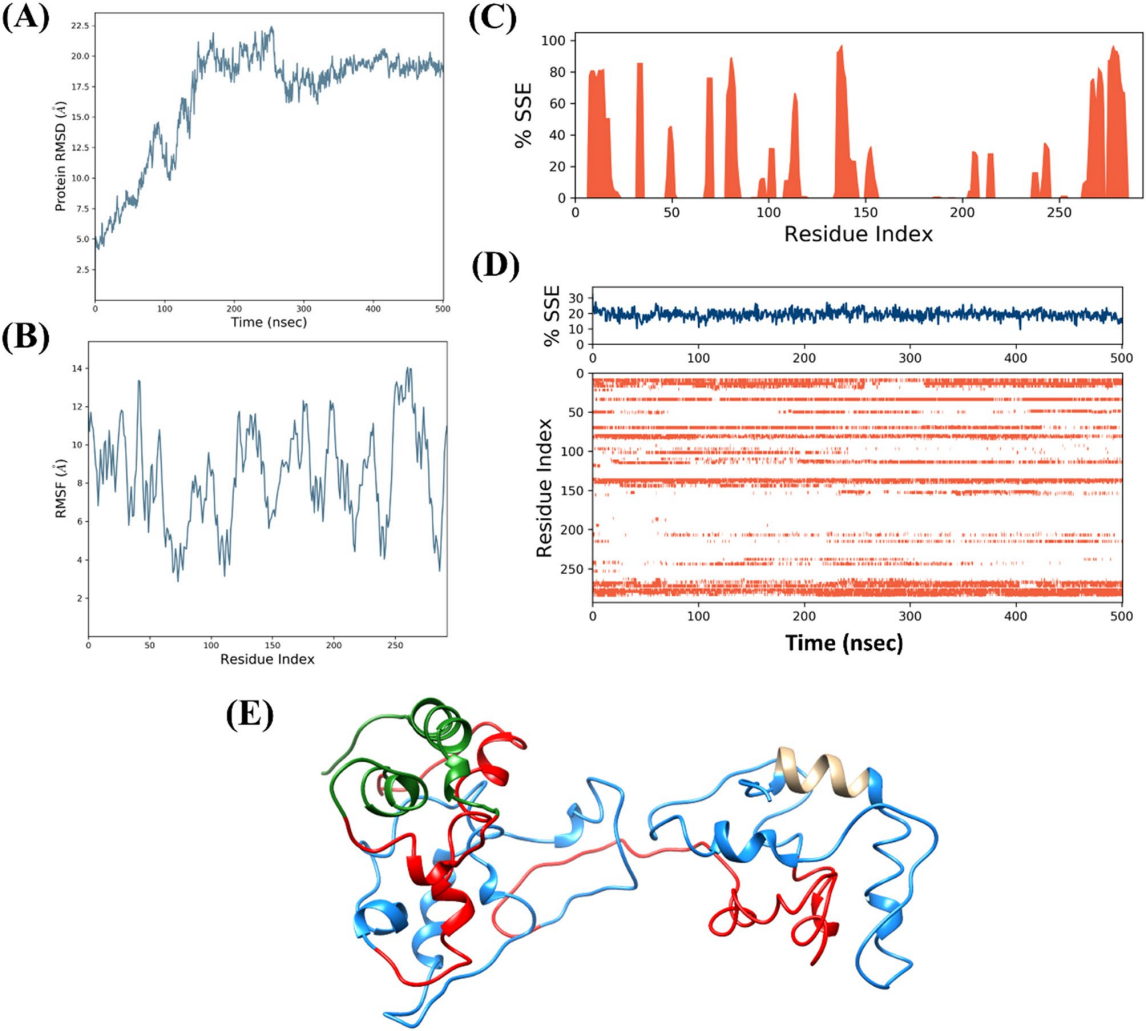
Molecular dynamic (MD) simulations analysis of Phosphoprotein (P): (**A**) root mean square deviation (RMSD), (**B**) root mean square fluctuation (RMSF)**,** (**C**) percentage secondary structure element (SSE; red color peaks show alpha helix) of phosphoprotein, (**D**) Timeline representation of secondary structure with respect to time, and (**E**) Last frame at 500 ns showing mostly unstructured regions after simulations. The disordered (IDPRs), MoRFs residues and MoRFs in IDP predicted regions are shown in red, tan and green colors, respectively.

In our prediction-based analysis, the P protein has been analyzed to be highly disordered among all CHPV proteins with approx. 50% of intrinsic disorder. Therefore, we have examined the structural dynamics using molecular dynamics simulations up to 500 ns of modeled 3D structure of P protein. The sequence-based protein BLAST result showed no similar structure in PDB that shows that no similar structure has been determined so far which may be due to its high disordered nature. Therefore, the threading approach of structure modeling (I-Tasser webserver) was employed. The modeled structure constituted a largely structured region with alpha-helix with some distorted geometry. After production MD run for 500 ns in an aqueous environment, the structure exposed several flexible regions and showed instability in the simulation. According to mean distances analyses at atomic level, the average RMSD of C-α atoms was approximately 17 Å which clearly explains the flexibility of a protein (Fig. 8A). The flexibility in the structure was also evident from hugely fluctuating RMSF values of P protein throughout the simulation period (Fig. 8B). In accordance with the atomic distances and fluctuation, the secondary structure element of P protein showed only ~ 19% after 500 ns (Fig. 8C). The same has been shown in Fig. 8D for each residue with respect to time. Lastly, the structural changes before and after simulations has been showed which depicts the transition of several helical regions to random coils (Fig. 8E).

## Conclusions

In this study, we present a new sphere of investigation that had remained unexplored in CHPV biology. We identified wide range of intrinsic disorder in all CHPV proteins, which may have a role in viral life cycle. We found that RNA-dependent polymerase L protein possesses the smallest level of intrinsic disorder and can be categorized as a highly ordered protein. On the other hand, the largest level of mean disorder is predicted in the phosphoprotein P, which is classified as a highly disordered protein in the CHPV proteome. We identified two disordered domains in phosphoprotein, which are hypothesized to have a critical role in function of this protein as a transcription-replication switch for the viral genome and therefore may be of particular interest. Additionally, we have supported our findings with extensive molecular dynamics simulation study. In MD simulations, the overall secondary structural composition was heavily reduced in comparison to the initial modeled structure. Furthermore, our MoRF analysis on the CHPV proteins predicted numerous disorder-based protein binding regions in all proteins. In many cases, for instance, phosphoprotein P, different predictor tools identify overlapping MoRF regions suggesting higher possibility and greater confidence of prediction. We expect this analysis to be helpful for understanding the ability of viral proteins to interact with their targets. Additionally, the position of predicted IDPRs and MoRFs are also shown in 3D structures of the CHPV proteins (which are crystal structures in case of G protein and models built using homology and threading based structure modelling). Such disordered and protein binding regions may play a number of important roles in viral pathogenicity, replication, host immune suppression, and viral particle assembly. Detailed experimental insights into functional disorder of viral proteins will help combat the viral spread and might have crucial implications for the design of drugs targeting disordered regions of viral proteins.

## Materials and methods

### Retrieval of CHPV protein sequences

The protein sequences of CHPV were retrieved from UniProt^96^. UniProt IDs for all five proteins are provided in the results and discussion sections of the individual proteins. We utilized these protein sequences for the prediction of disordered and disorder-based binding regions.

### Multiple sequence alignment (MSA)

The MSA was performed using Clustal Omega web server which generates alignments by utilizing Hidden Markov Model based techniques^97^. For enhanced visualization, the aligned sequences image processing was done using Esprit 3.0 server^98^.

### Evaluation of intrinsically disordered regions in CHPV proteins

The commonly used members of the Predictor of Natural Disordered Regions (PONDR) family were employed to predict intrinsic disorder in CHPV proteome. These include PONDR FIT^99–102^. Additionally, we used two forms of the IUPred2 tool^46^ (IUPred2 long and IUPred2 short) for the prediction of long and short IDPRs in CHPV proteins. We have also considered a predictor PrDOS which utilizes two different algorithms to compute the disorder scores. Based on support vector machine (SVM) algorithm and by analysing the conserved disordered regions of previously determined proteins, PrDOS produces the result with a cut-off of 0.5 (http://prdos.hgc.jp/cgi-bin/top.cgi). Residues with the disorder score values above 0.5 threshold values are considered as intrinsically disordered. The mean **p**redicted **p**ercent of **i**ntrinsic **d**isorder (PPID) was calculated for all five proteins from the outputs of all individual seven disorder predictors and the mean values as well. The PPID is calculated as

$${\text{PPID}} = \frac{{{\text{Number of residues with value }} \ge 0.{\text{5}}}}{{{\text{Total number of residues}}}} \times {\text{1}}00$$

For estimation of variability of individual predictors, we also calculated the standard deviation from all the data set of each predictor and to account for the variation in data from the mean, the standard error was calculated over mean values. The disordered regions were also predicted by MobiDB predictor containing MobiDB lite and other predictors (https://mobidb.bio.unipd.it/). It provides a consensus of several predictors to analyze disorderedness globally and also removes the chances of biased prediction of disorder regions.

### Molecular recognition features (MoRFs) prediction in CHPV

The web-based predictors were used to predict disordered-based protein binding regions/MoRFs. Each predictor uses a different set of algorithms for the prediction of MoRFs regions in the proteins. Thus, we used four different predictors such as MoRFCHiBi_Web (MCW; cutoff value 0.725)^103^, ANCHOR (0.5)^104^, MoRFpred (0.5)^105^, and DISOPRED3 (0.5)^106^. We have discussed the detailed methodology in our previous reports.

### Modeling of CHPV protein structures

The sequence based IDP predictions of proteins are quite more comprehensible with 3D structures. For CHPV proteins, there are two structures available for Glycoprotein (G) only. Therefore, we have modeled the full-length 3D structures for the remaining four proteins (L, N, M, and P). The modeling of CHPV N, M, and P protein structures were done by I-TASSER web-server, which utilizes the threading-based approach to construct a model^107^. However, the protein length limit for I-TASSER server is 1500 amino acids, whereas the L protein of CHPV is 2092 amino acid long. Therefore, we used Swiss-model^108^ to model L protein structure based on the homology to the template structures.

### Mapping of disordered and MoRF regions on modelled and available structures of CHPV proteins

The available structures of CHPV G protein were obtained from Protein data bank (PDB) and L, N, M, and P protein structures were modeled and used for mapping. The identified disordered and MoRFs regions were marked on the corresponding structures using UCSF Chimera. The colour schemes used to represent these regions on PDB, and Modelled structures are given in respective figure legends. The modeled structure was processed by adding missing hydrogen and assignment of proper bond orders to the structure in Schrodinger’s maestro. After preparation of structure, the simulation setup was built using TIP4P water model, neutralizing ions, and 0.15 M NaCl salt concentration. By utilizing Desmond simulation package, embedded in Schrodinger suite, we performed MD simulations using OPLS 2005 forcefield^109^. We have followed our previously used protocol for performing the simulations^110^.

## Supplementary Information

Supplementary Information 1.
