# Supplementary material for: Analysis of the dark proteome of Chandipura virus reveals maximum propensity for intrinsic disorder in phosphoprotein
Source: Sci Rep. 2021 Jun 24;11:13253. doi: 10.1038/s41598-021-92581-6 (PMC8225862; doi:10.1038/s41598-021-92581-6)
Supplement: Supplementary file 1 — Supplementary Information 1. [file 41598_2021_92581_MOESM1_ESM.docx]

**Supplementary Information**

**Analysis of the Dark Proteome of Chandipura Virus Reveals Maximum Propensity for Intrinsic Disorder in Phosphoprotein**

Nishi R. Sharma^1*^, Kundlik Gadhave^2^, Prateek Kumar^2^, Mohammad Saif^1^, Md. M. Khan^1^, Debi P. Sarkar^3^, Vladimir N. Uversky^4,5*^, Rajanish Giri^2*^

^1^School of Interdisciplinary Studies, Jamia Hamdard-Institute of Molecular Medicine (JH-IMM), Jamia Hamdard, Hamdard Nagar, New Delhi, 110062, India.

^2^School of Basic Sciences, Indian Institute of Technology Mandi, VPO Kamand, Himachal Pradesh, 175005, India

^3^Department of Biochemistry, University of Delhi South Campus, New Delhi, 110021, India

^4^Department of Molecular Medicine and Byrd Alzheimer’s Research Institute, Morsani College of Medicine, University of South Florida, Tampa, FL 33620, USA

^5^Institute for Biological Instrumentation of the Russian Academy of Sciences, Federal Research Center “Pushchino Scientific Center for Biological Research of the Russian Academy of Sciences”, Pushchino, 142290 Moscow, Russia

***Correspondence:** rajanishgiri@iitmandi.ac.in, vuversky@usf.edu, and nrsharma@jamiahamdard.ac.in


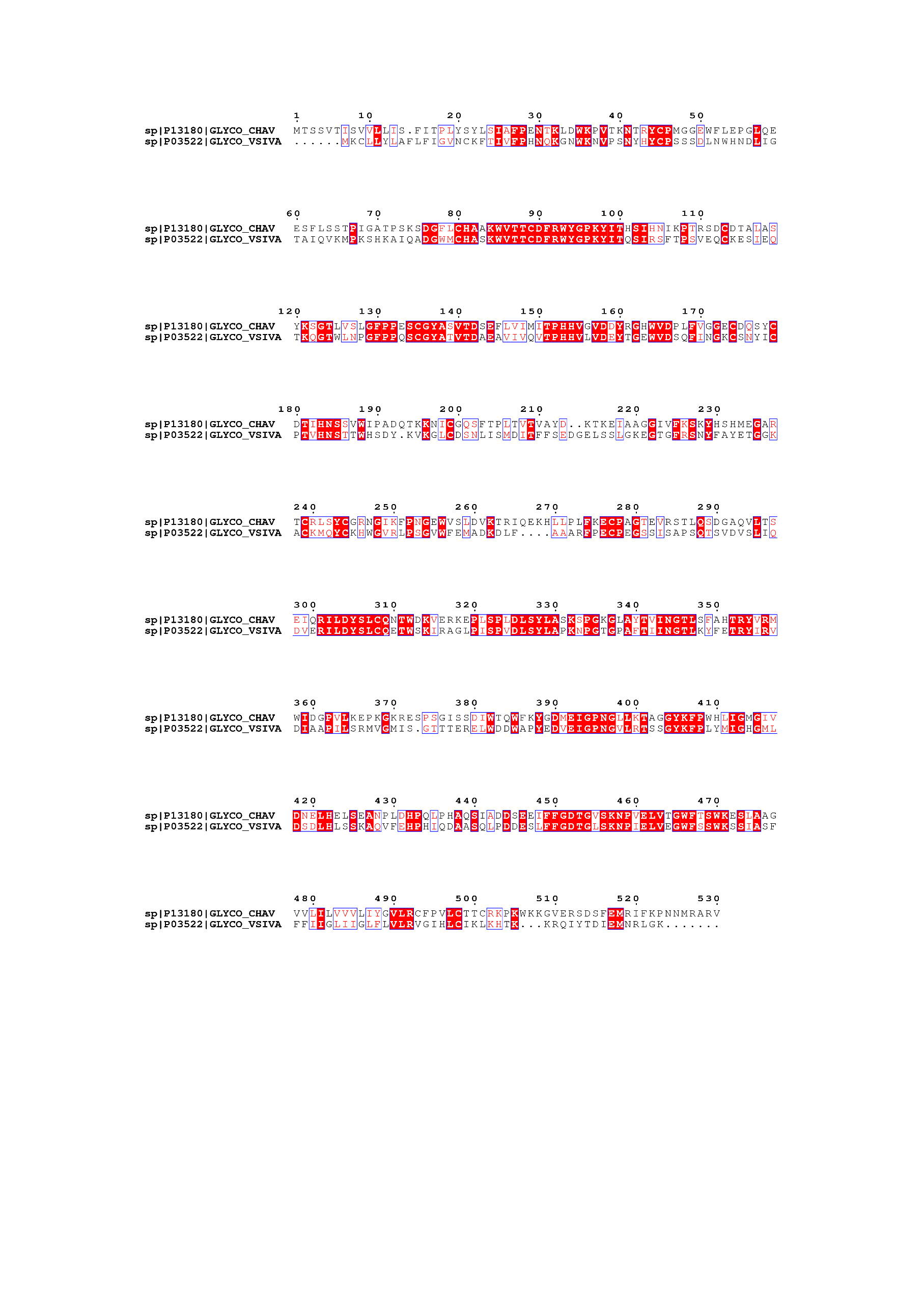


**Supplementary Figure 1:** Multiple sequence alignment analysis of Glycoproteins of Chandipura virus and Vesicular stomatitis Indiana virus (Uniprot ID: P03522).


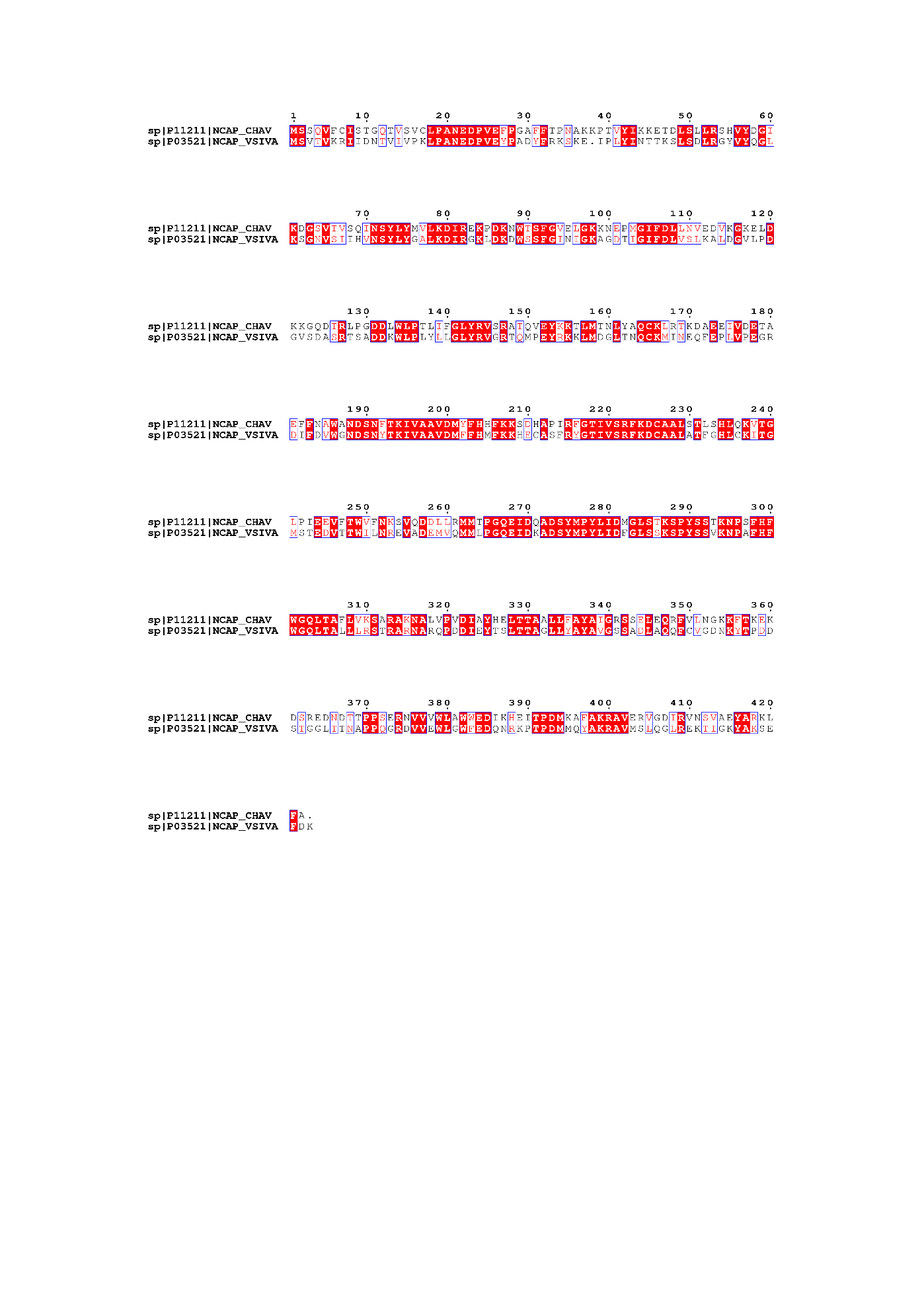


**Supplementary Figure 2:** Multiple sequence alignment analysis of Nucleoproteins of Chandipura virus and Vesicular stomatitis Indiana virus (Uniprot ID: P03521).

**Supplementary Figure 3:** Multiple sequence alignment analysis of RNA-directed RNA polymerase L (L) of Chandipura virus and Vesicular stomatitis Indiana virus (Uniprot ID: P03523).


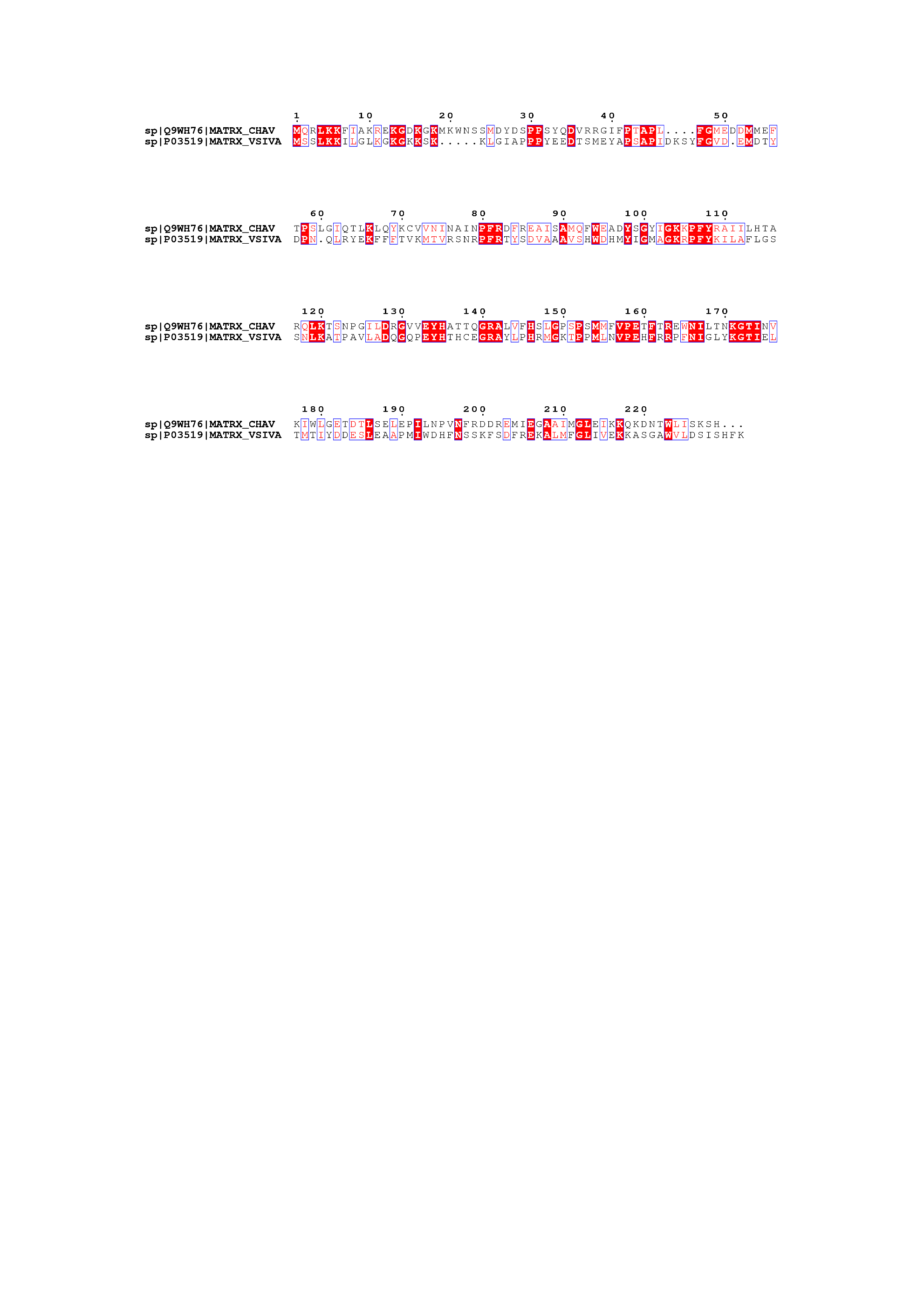


**Supplementary Figure 4:** Multiple sequence alignment analysis of Matrix of Chandipura virus and Vesicular stomatitis Indiana virus (Uniprot ID: P03519).


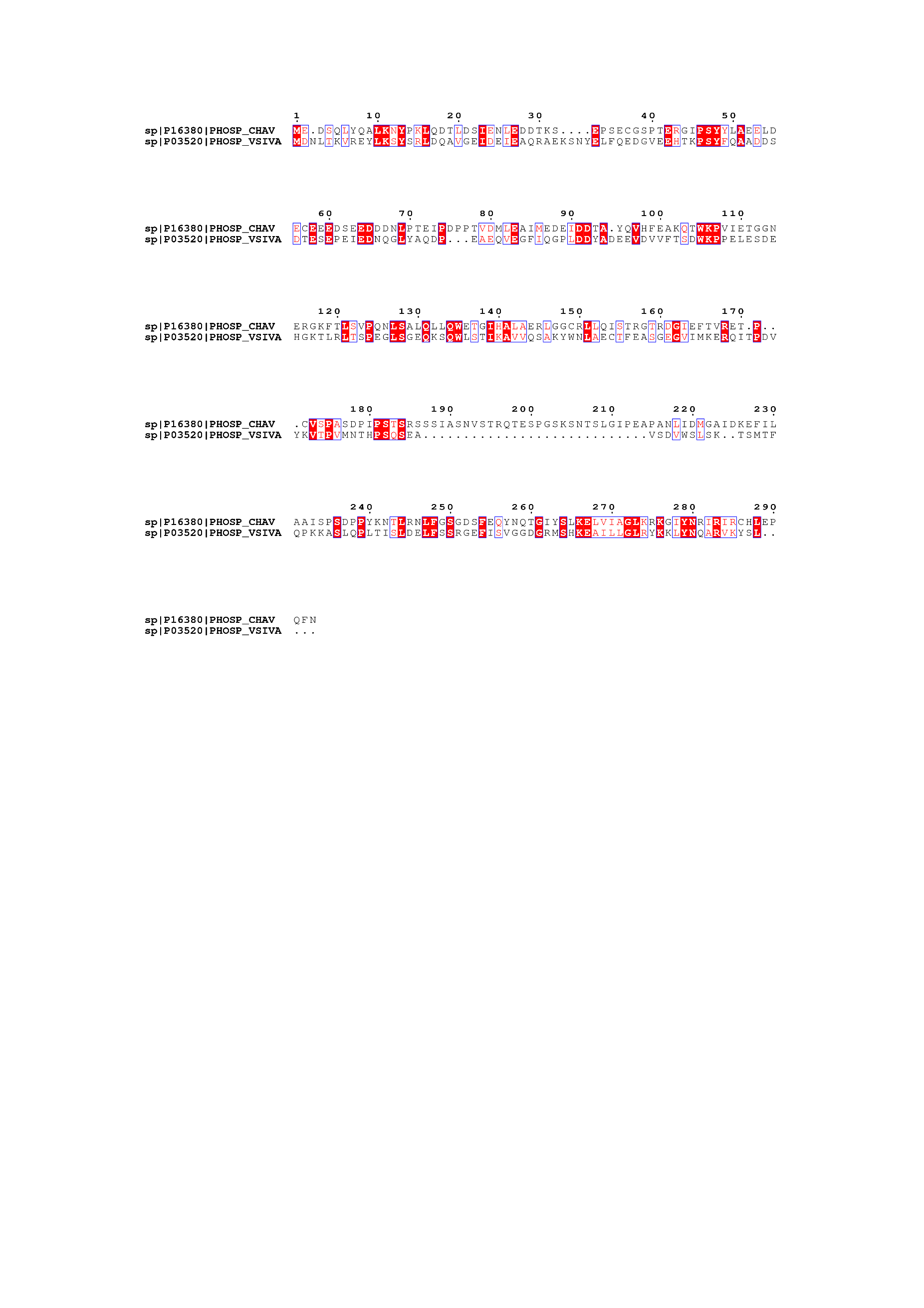


**Supplementary Figure 5:** Multiple sequence alignment analysis of Phosphoproteins of Chandipura virus and Vesicular stomatitis Indiana virus (Uniprot ID: P03520).
